# Developing a Phototactic Electrostatic Insect Trap Targeting Whiteflies, Leafminers, and Thrips in Greenhouses

**DOI:** 10.3390/insects12110960

**Published:** 2021-10-21

**Authors:** Yoshihiro Takikawa, Teruo Nonomura, Takahiro Sonoda, Yoshinori Matsuda

**Affiliations:** 1 Plant Center, Institute of Advanced Technology, Kindai University, Wakayama 642-0017, Japan; 2 Laboratory of Phytoprotection Science and Technology, Faculty of Agriculture, Kindai University, Nara 631-8505, Japan; nonomura@nara.kindai.ac.jp (T.N.); ymatsuda@nara.kindai.ac.jp (Y.M.); 3 Sonoda Seisakusho Co. Ltd., Osaka 547-0006, Japan; sonoda@ace.ocn.ne.jp

**Keywords:** attractive force, double-charged dipolar electric field, pest control, photoselective attraction, sticky trap, *Bemisia tabaci*, *Liriomyza sativae*, *Frankliniella occidentalis*

## Abstract

**Simple Summary:**

Silverleaf whiteflies (*Bemisia tabaci*), vegetable leafminers (*Liriomyza sativae*), and western flower thrips (*Frankliniella occidentalis*) are very serious pests of greenhouse tomatoes. In Japan, growers of organic tomatoes currently use large numbers of yellow sticky traps to control insects, but these traps need replacing very regularly, as the sticky surface becomes clogged with insects. An electric field-generating apparatus, described herein, is a potential physical tool to control these pests that have entered greenhouses. The electric field was formed in the space between oppositely electrified insulated conductors arrayed in parallel with fixed separation. Although these conductors created a sufficiently strong force to capture insects entering the field, the force was insufficient to capture insects outside the field. The positive phototaxis of these insects was an inspiration to develop an improved electrostatic insect trap, which was constructed by introducing oppositely charged yellow-colored water into paired transparent insulator tubes to produce opposite poles. The finished apparatus exhibited coloration and insect attraction characteristics similar to commercial yellow sticky traps, but had the advantage that they could be cleaned easily and remain effective for long periods. The surfaces of the insulator tubes containing the charged yellow water were electrostatically active, but not excessively sticky, thus, the apparatus could be placed close to the plants. The close location of the devices enabled preferential attraction of flying or plant-settling insects to the trap. The present study provided an experimental basis for developing an electrostatic device to attract and capture insects that enter greenhouses.

**Abstract:**

Our aim was to develop an electrostatic apparatus to lure and capture silverleaf whiteflies (*Bemisia tabaci*), vegetable leafminers (*Liriomyza sativae*), and western flower thrips (*Frankliniella occidentalis*) that invade tomato greenhouses. A double-charged dipolar electric field producer (DD-EFP) was constructed by filling water in two identical transparent soft polyvinyl chloride tubes arrayed in parallel with fixed separation, and then, inserting the probes of grounded negative and positive voltage generators into the water of the two tubes to generate negatively and positively charged waters, respectively. These charged waters electrified the outer surfaces of the opposite tubes via dielectric polarization. An electric field formed between the oppositely charged tubes. To lure these phototactic insects, the water was colored yellow using watercolor paste, then introduced into the transparent insulator tubes to construct the yellow-colored DD-EFP. This apparatus lured insects in a manner similar to commercially available yellow sticky traps. The yellow-colored DD-EFP was easily placed as a movable upright screen along the plants, such that invading pests were preferentially attracted to the trap before reaching the plants. Furthermore, pests settling on the plants were attracted to the apparatus, which used a plant-tapping method to drive them off the plants. Our study provided an experimental basis for developing an electrostatic device to attract and capture insects that enter greenhouses.

## 1. Introduction

A very serious problem in hydroponic cultures of greenhouse tomatoes is the entry of flying insect pests that pass through a conventional woven insect-proof net (mesh size: 1–1.5 mm). These pests frequently include whiteflies, *Bemisia tabaci* (Gennadius) (Hemiptera: Aleyrodidae), vegetable leafminers, *Liriomyza sativae* (Blanchard) (Diptera: Agromyzidae), western flower thrips, *Frankliniella occidentalis* (Pergande) (Thysanoptera: Thripidae), winged green peach aphids, *Myzus persicae* (Sulzer) (Hemiptera: Aphididae), fungus gnats, *Bradysia* spp. (Diptera: Sciaridae) and shore flies, *Scatella stagnalis* (Fallén) (Diptera: Ephydridae). Tomato plants are vulnerable to direct attacks by these pests, as well as serious infections caused by viral, bacterial, and fungal pathogens that are carried by these vectors. In particular, whiteflies, thrips, and aphids transmit viral pathogens (tomato yellow leaf curl virus [1,2], tomato spotted wilt tospovirus [3,4], and cucumber mosaic virus [5], respectively), whereas fungus gnats and shore flies transfer rhizosphere fungal pathogens (*Fusarium oxysporum* f. sp. *radicis-lycopersici* and *Verticillium dahliae*) [6,7]. To solve this issue, an electrostatic apparatus (electric-field screen) was devised, consisting of a grounded metal net parallel to a layer of negatively charged insulated iron wires that were arrayed in parallel with fixed separation [8]. This apparatus was installed in lateral windows of a greenhouse to repel insects that reached the metal net [9]. Notably, the apparatus was experimentally commercialized (Sonoda Seisakusho, Osaka, Japan) during 2017–2019 in Japan [10]. However, despite its great ability to repel insects, use of the apparatus among farmers was not extensive because of its high manufacturing and installation cost.

The photoselective behavior of insects has encouraged the exploration of colored sticky traps to examine population fluctuations of flying insects, either to facilitate control decisions in integrated pest management, or to evaluate experimental insecticide treatments in efficacy testing. Yellow sticky traps are effective at attracting many insect species, and they have been used to monitor populations of western cherry fruit flies, *Rhagoletis indifferens* (Loew) (Diptera: Tephritidae) [11], greenhouse whiteflies, *Trialeurodes vaporariorum*) (Westwood) (Hemiptera: Aleyrodidae) in greenhouses [12], *B. tabaci* and its parasitoid, *Eretmocerus emiratus* (Rose and Zolnerowich) (Hymenoptera: Aphelinidae) in fields [13], *B. tabaci* in greenhouses and fields [14], *F. occidentalis* in greenhouses [15], several thrips species in mango orchards [16], and American serpentine leafminers, *Liriomyza trifolii* (Burgess in Comstock) (Diptera: Agromyzidae) in greenhouses [17]. Similarly, blue sticky traps have been used to monitor populations of melon thrips, *Thrips palmi* (Karny) (Thysanoptera: Thripidae) [18] and bean flower thrips, *Megalurothrips usitatus* (Bagrall) (Thysanoptera: Thripidae) [19]. Recent analyses of the wavelength-specific behavior of insects revealed that thrips density was significantly suppressed in the presence of red light (600–660 nm) [18,20,21]. Based on these findings, Shimoda [22] noted the possibility that both red lighting and red-netting could be effectively used to control thrips species.

Almost all tomato growers in our district (Nara Prefecture, Japan) who cultivate the plants organically in large greenhouses use yellow sticky traps as an insecticide-independent method to reduce the populations of whiteflies and leafminers in greenhouses. For this purpose, many traps are hung from lateral pillars near the windows and crossbeams of a greenhouse—the traps are placed far from the cultivated plants because the trap surface is very sticky. However, the weakness of the trap is that the stickiness of its surface deteriorates gradually with an increasing number of trapped insects, therefore, farmers frequently exchange their traps for fresh traps during the peak season of pest appearance. The local farmers recently requested a less expensive and reusable trap with a non-sticky surface, which is able to attract and capture targeted insect pests.

The aim of our research is to develop physical measures to control insect pests. For this purpose, we devised some electrostatic apparatuses to create an electric field that generates an attractive force to capture insects [23].

Negatively and positively charged insulated conductors arrayed in parallel with fixed separation generate a static electric field (S-EF) in the space between them, where no discharge occurs [24]. This electric field is known as a double-charged dipolar (DD) electric field. An apparatus that generated a DD electric field was fabricated by paralleling a pair of iron wires (each was passed through a soft polyvinyl chloride (S-PVC) tube) with fixed separation, and linking those respective wires to the N- and P-VG. This DD electric field producer (DD-EFP) applied a strong attractive force to insects entering the field, such that they could not escape the trap [25].

Despite the great ability of the DD-EFP to capture insects, its limitation is that it cannot attract insects far from the apparatus. The purpose of the present study was to provide the DD-EFP with photoselective insect-attractivity, while preserving its strong insect-capturing ability. To solve this problem, the first experiment was designed to replace an iron conductor with colored water, and examine whether the water filled in the transparent S-PVC tube conducted sufficient electricity to generate negative or positive charges on the outer surface of the PVC tube via dielectric polarization. In the second experiment, a DD-EFP with yellow- or blue-colored S-PVC tubes was fabricated to examine its ability to capture test insects (whiteflies, western flower thrips, and vegetable leafminers) that were blown inside the electric field between the negatively and positively charged PVC tubes. In the last experiment, a yellow-colored DD-EFP was constructed as a movable upright object and placed beside potted tomato plants in a film house to attract remotely released insects (whiteflies and vegetable leafminers) to the DD-EFP. Based on the results obtained, we discuss the feasibility of the colored DD-EFP screen as a physical insect trap that can be fabricated easily and inexpensively from readily available materials.

## 2. Materials and Methods

### 2.1. Insects

Whiteflies *B. tabaci* (MEAM1) (Gennadius, 1889) [26], western flower thrips *F. occidentalis*, and vegetable leafminers *L. sativae* were used in the present study. Pupae of whiteflies and vegetable leafminers, and nymphs of western flower thrips, were purchased from Sumika Technoservice (Hyogo, Japan) and maintained in a growth chamber (25.0 ± 0.5 °C, 12-h photoperiod at 4000 lux) until use. Newly emerged adults of these insects were collected using an insect aspirator (Wildco, Yulee, FL, USA) for the experiments. The mean body sizes of the insects (i.e., length from head to wing tip, where 20 adults of each species were measured) were 0.81 ± 0.15 mm for the whiteflies, 1.42 ± 0.34 mm for the western flower thrips, and 2.04 ± 0.25 mm for the vegetable leafminers.

### 2.2. Electrification of S-PVC Tubes and Formation of the DD-Field for Insect Capture

**Figure 1:**
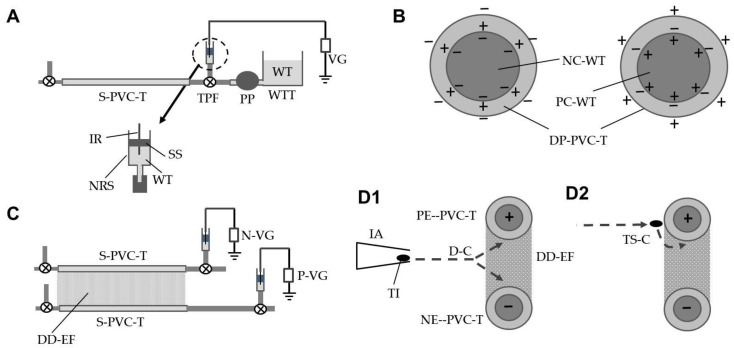
(**A**) Preparation of a soft polyvinyl chloride tube (S-PVC-T) filled with charged water. (**B**) Dielectric polarization of an S-PVC-T caused by the presence of negatively or positively charged water in the tube (cross-sectional view). (**C**) Formation of a double-charged dipolar electric field by a pair of oppositely charged PVC tubes. (**D1**) Insect capturing with the DD electric field (cross-sectional view) through a direct approach or (**D2**) a two-step approach. Dotted arrow depicts the path of an insect blown inside the DD electric field. Abbreviations: S-PVC-T, soft polyvinyl chloride tube; TPF, T-shaped acrylic pipe fitting with a channel-switching cock; PP, peristaltic pump; WT, water; WTT, water tank; VG, grounded voltage generator; IR, iron wire; NRS, needle-removed syringe; SS, silicone stopper; DP-PVC-T, dielectrically polarized PVC tube; NC-WT, negatively charged water; PC-WT, positively charged water; N-VG, negative voltage generator; P-VG, positive voltage generator; DD-EF, double-charged dipolar electric field; PE-PVC-T, positively electrified PVC tube; NE-PVC-T, negatively electrified PVC tube; IA, insect aspirator; TI, test insect.

A transparent S-PVC tube (wall thickness, 0.5 mm; inner diameter, 3 mm; resistivity, 10^9^ Ω·cm) (Junkosha Inc., Tokyo, Japan) was used to construct the DD-field. Both ends of the S-PVC tube (length, 100 m) were connected with T-shaped pipe fittings using a channel-switching cock—the two open ends of the fitting on one side were linked to a water tank and a needle-removed injector syringe (inner diameter, 1 cm; length, 5 cm) with a silicone stopper, respectively. The silicone stopper was pierced by an iron wire (diameter, 0.1 mm; length, 3 cm) (Figure 1A). Water in the tank was sent to the S-PVC tube and syringe using a peristaltic pump. After the tube and syringe had been filled with water, the pipe fittings on both ends were closed, and the pump and water tanks were then removed. The end of the iron wire outside the syringe was connected to a negative or positive voltage generator (Max Electronics, Tokyo, Japan) to negatively or positively electrify the S-PVC tube.

An electric field is generated in the space surrounding a charged conductor, where the charged conductor can exert strong attractive or repulsive force against other charges in the field [27]. A conductor (e.g., iron wire or plate, as in our previous studies) is charged with a grounded negative or positive voltage generator. The grounded negative voltage generator (N-VG) draws free electrons (negative charges) from the ground that accumulate on the conductor surface (i.e., negative charging of the conductor), and a positive voltage generator (P-VG) pushes free electrons from the conductor to the ground, leaving it positively charged (i.e., positive charging of the conductor). Using an insulated conductor linked to the N-VG, the negative charge on the conductor surface is not released in the electric field, and the conductor connected with the N-VG receives no electricity from the electric field. Negative or positive charges on the conductor surface cause dielectric polarization in the insulating coating, thus, negatively and positively electrifying the outer surface of the insulator coating, respectively [28]. These surface charges create an electric field that surrounds the insulated conductor.

Figure 1B schematically illustrates the electrification of the S-PVC tube via dielectric polarization, which was caused by negatively or positively charged water in the tube and syringe. The negative charge of the water created dielectric polarization within the tube, thereby negatively electrifying the outer surface of the S-PVC tube. Similarly, positively charged water positively electrified the tube outer surface.

Water was colored yellow or blue using watercolor pastes (Turner Color Works Ltd., Osaka, Japan), and their Munsell hue/value/chroma indexes [29] were 7Y8.5/11 (yellow) and 7.5PB3/10 (blue), which corresponded to the coloration of commercially available yellow and blue sticky traps (Horiver yellow and blue traps; Arysta LifeScience Corp., Tokyo, Japan). The S-PVC tube was filled with water in which different weights of watercolor paste had been dissolved, and the coloration of the tube was measured using an RGB-1002 color analyzer (Sato Shoji Inc., Kanagawa, Japan). The colorant concentration of 750 mg per 100 mL of water, which yielded the yellow and blue indexes mentioned above, was used to color the DD-FP water. The same concentration was used to make a red-colored DD-FP, and the index (Munsell 7R5/14) was identical to the index of a commercial red insect-proof net (Sunsun-net e-red; Miekounousha, Mie, Japan).

Surface charges of tubes containing non-colored and colored water were measured at different locations from the tube end (site of charging) using an FMX-002 electrostatic field meter (Simco, Kobe, Japan) to determine changes in potential difference (with respect to earth ground) at each location.

Figure 1C illustrates the generation of the DD-field using oppositely electrified S-PVC tubes. The tubes were horizontally arrayed in parallel, 5 mm apart, and the water in the tubes was charged with equal, but opposite, voltages. The DD-field formed in the space between the opposite charges of the tubes (Figure 1D).

An insect-capturing assay was conducted at the region located 0.9–1.1 m from the tube end (charging side). The ability of the DD-field to capture insects was examined by blowing test insects (whiteflies, thrips, and leafminer adults) toward the space between two S-PVC tubes using an insect aspirator (Figure 1D). The tubes were oppositely charged with different voltages to determine the lowest voltage that could capture all insects blown inside the DD-field. Twenty insects were used for each species. Insects were captured by the nearest tube. The mechanism for insect capture was described previously [25]. Similar experiments were conducted at other regions (19.9–20.1 and 29.9–30.1 m from the tube end) when charged at ± 5.5 kV, which was the lowest voltage that could capture all insects.

### 2.3. Construction of the DD-EFP

**Figure 2:**
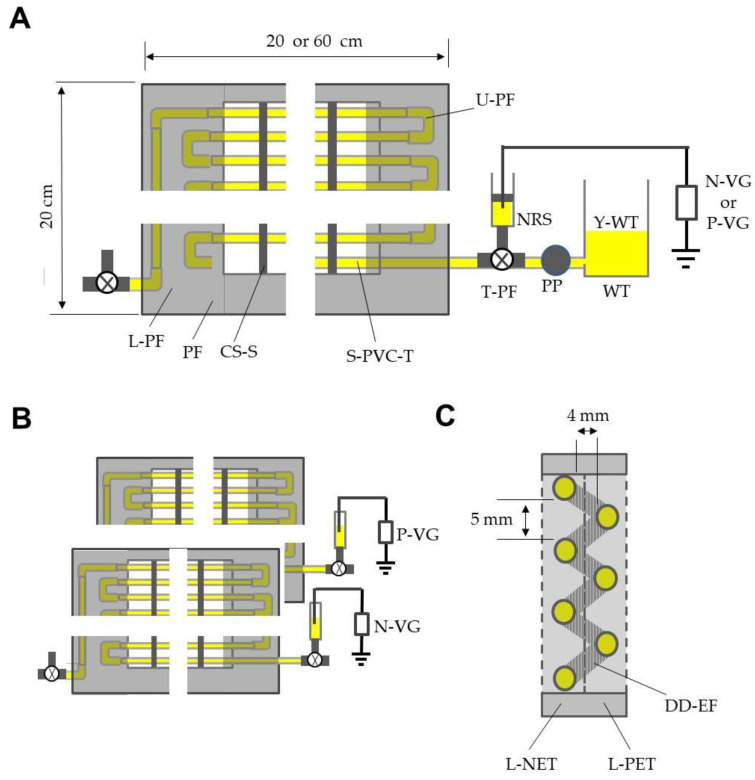
(**A**) A layer of soft polyvinyl chloride (S-PVC) tubes electrified with negatively or positively charged yellow-colored water. (**B**) Two layers of S-PVC tubes oppositely electrified with charged water. (**C**) Construction of the double-charged dipolar electric field producer (DD-FP), which was constructed by coupling two layers of S-PVC tubes that had been oppositely electrified (cross-sectional view). Abbreviations: L-PF, L-shaped pipe fitting; PF, polypropylene frame; CS-S, comb-shaped polypropylene spacer; S-PVC-T, soft polyvinyl chloride tube; U-PF, U-shaped pipe fitting; NRS, needle-removed syringe; T-PF, T-shaped pipe fitting; WT, water tank; PP, peristaltic pump; Y-WT, yellow-colored water; N-VG, negative voltage generator; P-VG, positive voltage generator; L-NET, layer of negatively electrified tubes; L-PET, layer of positively electrified tubes; DD-EF, double-charged dipolar electric field.

Figure 2A schematically illustrates the structure of the S-PVC tube layer. Twenty S-PVC tubes (15 cm long) were horizontally held in parallel, separated by 5 mm using four comb-shaped polypropylene spacers. The ends of the tubes were linked to each other with U-shaped pipe fittings and fixed to a polypropylene frame to make a layer of tubes. The open ends of the lowest and highest tubes were connected with T-shaped pipe fittings that had been equipped with channel-switching cocks. Both open ends of the fitting on one side were similarly connected to a water tank, and a negative or positive voltage generator. In this figure, as an example, the tubes and syringe were filled with yellow-colored water by means of a peristaltic pump linked to the water tank, and then, the pump and tank were removed. Two identical tube layers (20 × 20 cm^2^) were coupled to make the DD-EFP (Figure 2B). Tubes of the layers were offset, such that the DD-field formed between all tubes (Figure 2C). Two height-adjustable legs were attached to the DD-EFP. In the following experiment, the tubes of two layers were oppositely charged at ± 1.2 kV, which was the lowest voltage that could capture all insects entering the DD-field.

### 2.4. Assay of the Ability of Colored DD-EFP to Attract Insects

**Figure 3:**
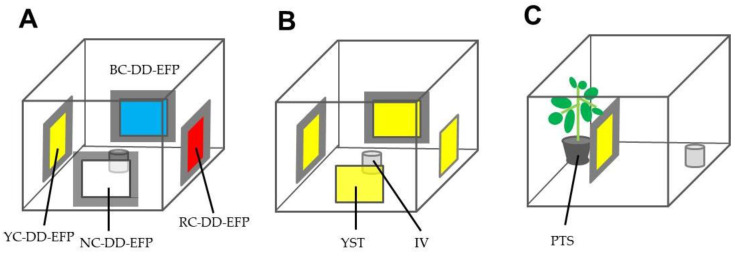
Experimental design to test the abilities of colored and non-colored double-charged dipolar electric field producers (DD-EFPs) to attract test insects (adults of whiteflies, western flower thrips, and vegetable leafminers). (**A**): Yellow- (YC-DD-EFP), blue- (BC-DD-EFP), red- (RC-DD-EFP), and non-colored (NC-DD-EFP) apparatuses were placed in the cubic transparent acrylic box and along four lateral faces of the box; an open vial containing test insects was placed at the center of the box. (**B**): Two YC-DD-EFPs and two sticky traps (YST) were similarly placed in the box. (**C**): Potted tomato seedling (PTS) (30 cm high (from pot bottom to plant tip)) and an insect vial (IV) were placed along the opposite faces of the box, and a YC-DD-EFP was placed in front of the plant. In all experiments, test insects were tracked for 2 h after their release.

In the first experiment, the four types of DD-EFPs (20 × 20 cm^2^; yellow-, blue-, red-, and non-colored) were placed in a transparent acrylic box (50 cm^3^) and along four lateral faces of the box, and a non-capped 2-mL vial containing test insects was placed at the center of the box (Figure 3A). The box was illuminated for 12 h by a white fluorescent lamp (25.2 μmol m^−2^s^−1^) that was located 30 cm above the roof of the box.

In the second experiment, two yellow-colored DD-EFPs and Horiver yellow sticky traps (20 × 20 cm^2^) were placed along the opposite faces of the box (Figure 3B). An insect-containing vial was placed at the center of the box.

In the third experiment, a potted tomato seedling (1 month old, 40 cm high (from pot bottom to plant top)) and an insect vial were placed along the opposite faces, and a yellow-colored DD-EFP was placed in front of the potted plant (Figure 3C).

In all experiments, at 24 h after the insects were released, we counted the number of test insects that had been captured by the DD-EFPs and sticky trap plates, the number of test insects that had reached the plant, and the number of test insects that remained in the vial or on the box walls and ceiling. Twenty insects were used for each insect species. Experiments were separately conducted using single insect species, and repeated five times for each species.

### 2.5. Simplification of DD-EFP Insect-Capturing System for Practical Use

**Figure 4:**
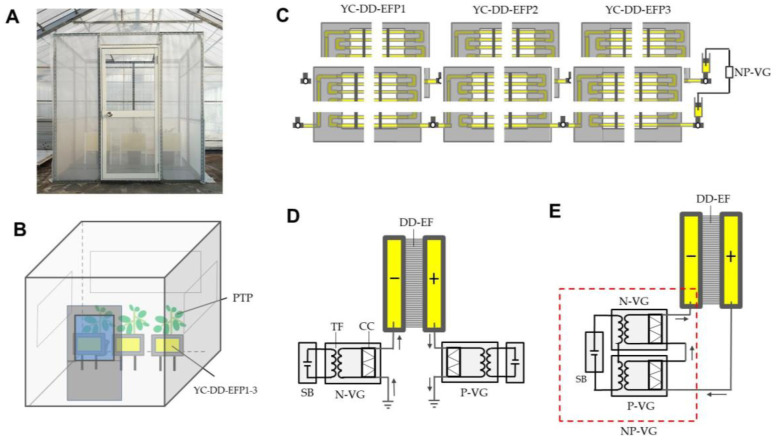
(**A**) Polyvinyl chloride-sheeted film house (2 m^3^) placed in a greenhouse, where an insect attraction assay was conducted. (**B**) Three potted tomato plants (2 months old, 1 m high) and triplicated yellow-colored double-charged dipolar electric field producers (YC-DD-EFPs) placed in front of plants in the film house. (**C**) Triplicated YC-DD-EFP1 3 placed in front of the plants. (**D**,**E**) Schematic representations of grounded (**D**) and non-grounded (**E**) circuits integrated into the voltage generator. Arrow indicates the direction of movement of negative electricity or free electrons. Abbreviations: PTP, potted tomato plants; YC-DD-EFP1 3, triplicated YC-DD-EFPs; DD-EF, double-charged dipolar electric field; TF, transformer; CC, Cockcroft circuit; SB, storage battery; P-VG, positive voltage generator; N-VG, negative voltage generator; NP-VG, negative and positive voltage generators unified in a box.

An insect attraction assay was conducted using a PVC-film-covered 2 × 2 × 2-m^3^ cubic house that had been placed inside a glass house (Figure 4A) to trace the behavior of the insects released, and the diurnal temperature change in the film house was between 16 °C and 32.8 °C. In this experiment, three potted tomato plants (2 months old, approximately 1 m high (from pot bottom to plant top)) were placed along one face of the closed film house, and three yellow-colored DD-EFPs of larger size (60 × 20 cm^2^) were constructed and placed in front of the plants (Figure 4B). Three identical tube layers were linked to each other with T-shaped pipe fittings, as noted above. Two sets of triplicated tube layers were placed facing each other (Figure 4C), and the water in the tubes in each set was negatively and positively charged using a non-grounded voltage generator (±1.2 kV fixed type) (Max Electronics, Tokyo, Japan), in which negative and positive voltage generators were unified in a box [30] (Figure 4D,E). Note that two grounded voltage generators were not used in this design. The voltage generator was operated by a 12-V rechargeable storage lithium battery. In this experiment, whiteflies and vegetable leafminers were tested.

In the first experiment, an insect aspirator containing two types of insects (20 insects per species) was inserted into a small hole of the door, and all insects were simultaneously blown inside the film house. After 2 h of blowing, the numbers of insects that were captured by the DD-EFPs, insects settled on tomato leaves, and insects remaining on other surfaces (the floor, walls, and ceiling of the cabinet) were counted.

In the second experiment, both test insect species (20 insects per species) were randomly transferred onto leaves of three tomato plants using an insect aspirator. After all of the insects had settled on the plant leaves, the DD-EFPs were charged at ± 1.2 kV, and the plants were then gently tapped by hand to force the insects to fly from the plants. Tapping was performed once or 2–5 times at intervals of 30 s. After 30 min of tapping, the number of insects captured by the DD-EFPs was counted.

In the last experiment, insects were similarly blown inside the film house, and 1 h later, the plants were tapped five times using the method described above. The number of insects captured by the DD-EFPs was counted at 1 h after tapping.

### 2.6. Statistical Analysis

All experiments were repeated five times, and data are presented as means ± standard deviations. Using EZR software version 1.54 (Jichi Medical University, Saitama, Japan), Tukey’s test was performed to identify significant differences among conditions, as indicated in the figure and table legends

## 3. Results and Discussion

### 3.1. Electrification of an Insulator Tube by Charged Colored Water

The first aim of the present study was to confirm that water filled in the S-PVC tube could be charged by touching it with the probe tip of a voltage generator. After the water had become charged, the S-PVC tube surrounding the water should have been electrified via dielectric polarization. Eventually, the electrified tube surface would generate a potential difference to the earth ground, which is theoretically equivalent to the absolute value of the voltage applied to the conductor [28]. With respect to the long tube (i.e., long path of water), however, it is essential to examine the reduction of potential difference at various locations distant from the tube end (the site at which the water is charged) because current density decreases with decreasing electrical conductivity (expressed in Siemens per meter) [31]. Table S1 lists the absolute values of the potential difference measured at various locations along S-PVC tubes in which the water was filled and charged at different voltages, in the absence and presence of watercolor. In the watercolor-free water, a reduction in potential difference was initially detected at 40 m from the tube end, and it increased with increasing distance. A similar result was obtained regarding S-PVC tubes that had been electrified with the differently colored water, which confirmed that the colorants did not affect water conductivity. In all test conditions, the reduction of potential difference was very small, and its effect on force generation (discussed below) was considered negligible.

### 3.2. Construction of the DD-EFP for Capturing Insects

**Figure 5:**
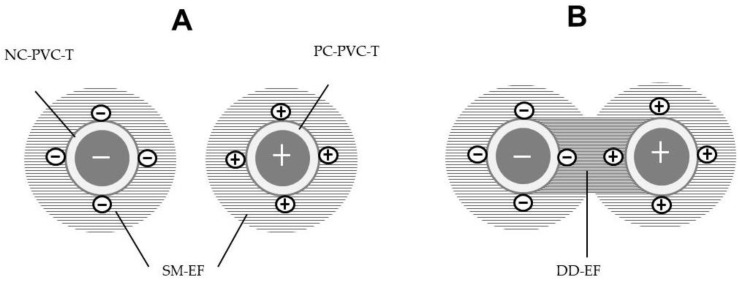
(**A**) Single-charged monopolar and (**B**) double-charged dipolar electric fields formed by negatively and positively electrified soft polyvinyl chloride tubes that were filled with oppositely charged water. Abbreviations: NC-PVC-T, negatively charged polyvinyl chloride tube; PC-PVC-T, positively charged polyvinyl chloride tube; SM-EF, single-charged monopolar electric field; DD-EF, double-charged dipolar electric field.

A voltage generator is a booster that increases an initial voltage (12 V) to a desired voltage (1–10 kV) by means of a Cockcroft circuit integrated in the generator [32]. The enhanced voltage is used to transfer negative electricity from/to the conductor connected to the voltage generator. Negatively and positively charged conductors accumulate negative and positive charges, respectively, on their outer surfaces. When an insulated conductor is present, negative and positive charges on the conductor surface cause respective negative and positive electrifications of the outer surface of the insulating coating via dielectric polarization [28]. In this experiment, the negatively and positively electrified S-PVC tubes created a single-charged monopolar electric field (SM-field) around tubes with the same field strength (Figure 5A), where the force to capture insects was generated. However, the force was not sufficiently strong to continuously capture insects with the tube. A stronger force was created by placing oppositely electrified tubes in parallel, separated at a fixed distance [24]. Figure 5B illustrates a DD-field produced between oppositely electrified PVC tubes. The DD-field is double the potential difference of the SM-field. The field strength of the DD-field is determined by the distance between the opposite pole (pole distance) and the voltage applied to the poles—shorter distances and larger voltages create stronger field strength (i.e., stronger force to capture insects that enter the DD-field) [33]. In the present study, the pole distance was fixed at 5 mm, and the negative and positive voltages applied to the poles were adjusted to determine the lowest voltage that could capture all insects that had been blown inside the DD-field.

Matsuda et al. [25] explained the movements of vinegar flies (*Drosophila melanogaster*) that had been released in a DD-field, which was formed by oppositely electrified PVC tubes into which charged iron conductor wires were inserted. In this field, the insects that were released near the negatively electrified insulated conductor (negative pole) were deprived of negative charge and became net positive, leading to direct attraction to the negative pole. In contrast, insects released near the positive pole were charged with free electrons localized around the positive pole, and they became net negative and were drawn toward the positive pole. In the present study, a DD-EFP was constructed using S-PVC tubes that had been charged with water, instead of a metal conductor, and this DD-EFP was examined for its ability to capture test insects. Table 1 lists the rates of test insects (adults of whiteflies, western flower thrips, and vegetable leafminers) captured by oppositely electrified S-PVC tubes in which non-colored and colored (yellow, blue, and red) water had been filled and charged at different voltages. Table 1 lists the results at 0.9–1.1 m from the tube end where the voltage generator probe was touched. The force generated in the DD-field increased with increasing applied voltage. Higher voltages were required to capture insects with larger body sizes (whiteflies < western flower thrips < vegetable leafminers). At ±1.2 kV, all insects blown into the field were captured strongly by either S-PVC tube, such that they could not escape from the trap. Video S1A shows the direct capture of test insects (whiteflies) by the yellow-colored DD-EFP as they were blown into the electric field (Figure 1D1). Notably, Video S1B shows that insects trapped at the front of the tube were pulled inside the DD-field, where they could be captured more tightly, through a two-step capturing process (Figure 1D2). There were no differences in capturing ability between non-colored and colored tubes. At lower voltages, although the insects became trapped, they were able to struggle and escape the tube. Accordingly, the DD-EFP was charged at ±1.2 kV for subsequent experiments.

The insect-capturing assay was conducted at tube surface locations varying in potential difference, and tubes were oppositely charged at ±1.2 kV. All insects could be captured by the oppositely electrified tube pair, regardless of location and test insect. We concluded that the decreasing potential difference with increasing distance from the charging tube end had no effect on insect-capturing ability.

### 3.3. Addition of Insect-Attracting Ability to the DD-EFP

**Figure 6:**
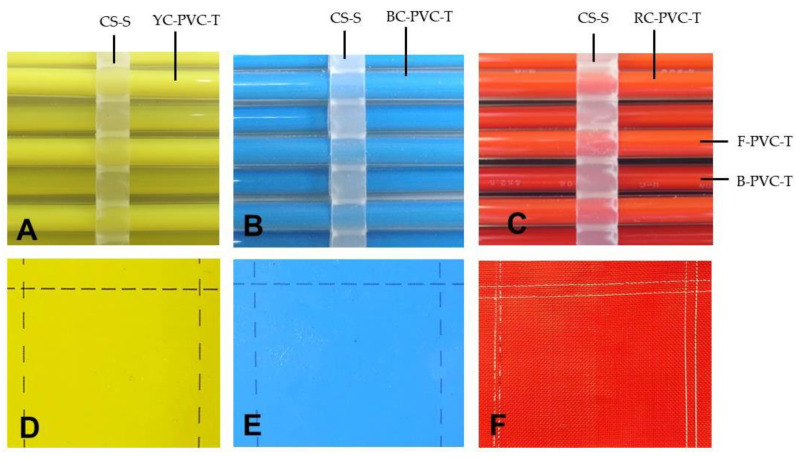
Comparison of hue-/value-/chroma-based coloration among transparent PVC tubes containing (**A**) yellow-, (**B**) blue-, and (**C**) red-colored water, and commercially available (**D**) yellow and (**E**) blue sticky traps, or (**F**) a red insect-proof net. Abbreviations: CS-S, comb-shaped polypropylene spacer; YC-, BC-, and RC-PVC-T, transparent PVC tube containing yellow-, blue-, and red-colored water, respectively; F- and B-PVC-T, front and back PVC tubes of a paired tube layer, respectively.

The present study demonstrated that the DD-EFP had sufficient ability to capture insects that entered the electric field. Hence, the next step was to provide the DD-EFP with the ability to lure insects distant from the apparatus. Nonomura et al. [24] constructed an electrostatic apparatus using oppositely charged insulated metal wires that had been arrayed alternately and placed in front of a yellow board. This attracted and captured flying adults of greenhouse whiteflies because of their phototactic response to yellow. In the present study, we focused on adding an insect-attracting ability to the DD-EFP to control phototactic insect pests, such as whiteflies and vegetable leafminers. Coloring the apparatus was the primary step for this purpose. Our initial goal was to assemble colored S-PVC tubes that exhibited suitable bulk resistivity (approximately 10^9^ Ω·cm), which is essential for PVC tubes to exhibit a strong insect-capturing function [34]. Unfortunately, such materials were not commercially available, therefore, we selected an alternative method: the introduction of colored water into the transparent PVC tube. The advantage of this method was the easy adjustment of the hue/value/chroma of the water in the tube. In the present study, watercolor pastes from Turner Color Works Ltd. were used as colorants to tint the water to the same color as commercial yellow and blue sticky plates (Figure 6A,B, respectively), or a red insect-proof net (Figure 6C). The concentration of the watercolor paste was adjusted to ensure that the reflected color of the S-PVC tube matched the colors of the yellow and blue sticky plates, or the color of the red net. Hereafter, colored DD-EFPs prepared by this method were used for insect attraction assays.

**Figure 7:**
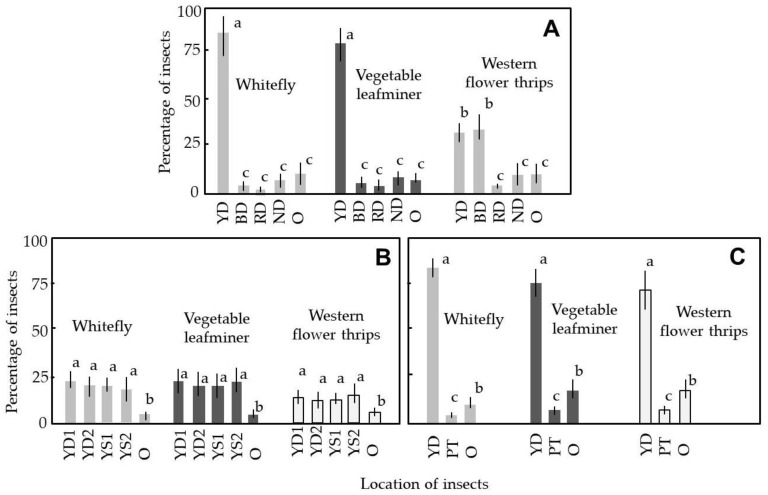
(**A**) Comparative assay of insects’ photoselective responses to three colored double-charged dipolar electric field producers (DD-EFPs) and a non-colored DD-EFP. Four DD-EFPs were placed in the closed box and along four faces of the box; an open vial containing test insects was placed at the center of the box. (**B**) Comparison of attraction of test insects between yellow-colored DD-EFPs and sticky traps. Two DD-EFPs and sticky traps were placed along four faces of the box; an insect-containing vial was placed at the center of the box. (**C**) Assay of preferential attraction of test insects by a yellow-colored DD-EFP placed in front of a potted tomato plant. A potted plant and insect vial were placed on opposite sides of the box, respectively; the DD-EFP was placed in front of the plant. Adult insects (whiteflies, vegetable leafminers, western flower thrips) were used as test insects. In all experiments, destinations of test insects were recorded 2 h after their release. YD, BD, RD, and ND represent yellow-, blue-, red-, and non-colored DD-EFPs, respectively; O represents other places, such as in the vial or on the floor, wall, and ceiling of the box. YD1 and YD2, and YS1 and YS2, correspond to two yellow-colored DD-EFPs and yellow sticky traps, respectively; PT indicates a potted tomato plant. Twenty insects of each species were used in each experiment; the means and standard deviations were calculated from five replicates of the experiments. The letters (a–c) in each vertical column indicate significant differences (*p* < 0.05) according to Tukey’s test.

Figure 7 shows the relative ratios of test insects with phototactic responses to the colored DD-EFPs or sticky traps that had been placed in a closed box. In the first experiment, we examined whether these phototactic insects showed photoselective responses to different colors of the DD-EFPs (Figure 7A). The results indicated that whiteflies and vegetable leafminers were preferentially attracted to the yellow-colored DD-EFP, whereas western flower thrips were preferentially attracted to both yellow- and blue-colored DD-EFPs equally. These responses were in complete agreement with the expected behaviors for these phototactic insects [35], which indicated that the colored DD-EFPs constructed using the present method were effective for attracting phototactic insects. In contrast, few test insects of any type were captured by the red-colored DD-EFP, and these relative ratios were not significantly different from the relative ratios of insects that were captured by the non-colored DD-EFP, or the insects that remained at other places. Shimoda [22] noted that many insects are unable to see red light (600–700 nm). We attribute the insect capturing by the red-colored DD-EFP to accidental contact of the insect with the apparatus, rather than to attractive capturing.

In subsequent experiments, the yellow-colored DD-EFP was used because of its ability to attract all types of test insects. Figure 7B shows that there were no significant differences in the numbers of captured insects between the yellow-colored DD-EFP and the yellow sticky trap, which indicated that the yellow-colored DD-EFP was functionally equal to the commercial yellow sticky trap. The results presented in Figure 7C depict a significant difference in the number of insects trapped between the yellow-colored DD-EFP and the number of insects on plants placed behind the DD-EFP. These findings indicate that the yellow-colored DD-EFP located in front of the plants was able to preferentially attract test insects, thereby effectively minimizing the number of insects visiting host plants.

The wide selection of commercially available watercolors facilitates the achievement of any desired water coloration. We leveraged this accessibility to fabricate yellow-, and blue-colored DD-EFPs, which were able to attract phototactic insects distant from the apparatus. The colored DD-EFP is useful as a trap and as an experimental tool to analyze the photoselective behavior of various insects. Indeed, large variations in DD-EFP coloration are readily achieved, and these would be suitable for an extensive analysis of responses by a large range of insect species. Moreover, it is possible to subtly change yellow or blue colors via different combinations of coloring factors (hue, value, and chroma), such that the difference in color response of a phototactic insect species may be investigated according to the color index. We expect future research to investigate these experimental designs.

### 3.4. Feasibility of the DD-EFP for Practical Use

Prior to conducting an insect-capturing assay in a film house, we modified the present DD-EFP system for practical use. The overall configuration of the present DD-EFP was simple, therefore, it was easy to fabricate at different scales. In the present study, however, instead of manufacturing a large-sized DD-EFP, we fabricated considerably smaller, connected individual DD-EFPs to provide an equivalent large size—this assembly was easier and could be more effectively regulated. Additionally, we replaced two grounded voltage generators (negative and positive) with a non-grounded and battery-operated voltage generator [30]. Thus, the DD-EFP system could be used without setting up a grounded line and electric power supply. The grounded negative voltage generator draws negative electricity from the ground and supplies it to the linked conductor, and the grounded positive voltage generator sends the electricity of the conductor to the ground (Figure 4D). In contrast, a non-grounded voltage generator sends the electricity of the conductor to the negative voltage generator, which supplies another linked conductor (Figure 4E)—this type of voltage generator does not require a ground line. Accordingly, there was no limitation on placement of the DD-EFP.

The DD-EFP can be fabricated using commonly available materials. The only electric part that must be purchased is the voltage generator. Two configurations (voltage-fixed and voltage-adjustable) voltage generators are commercially available. The voltage-adjustable type, which is generally more expensive, was used to determine the optimal voltage condition during our investigation, and the voltage-fixed type was applied to the practical apparatus because of its lower cost.

The present study demonstrated the outstanding ability of the DD-EFP to capture and retain insects. The insects died within 3–6 h of capture (data not shown). The present apparatus generated an electrostatic force, therefore, its surface was not sticky at all. This highly convenient feature enabled the removal of captured insects by simply using a waterjet.

**Figure 8:**
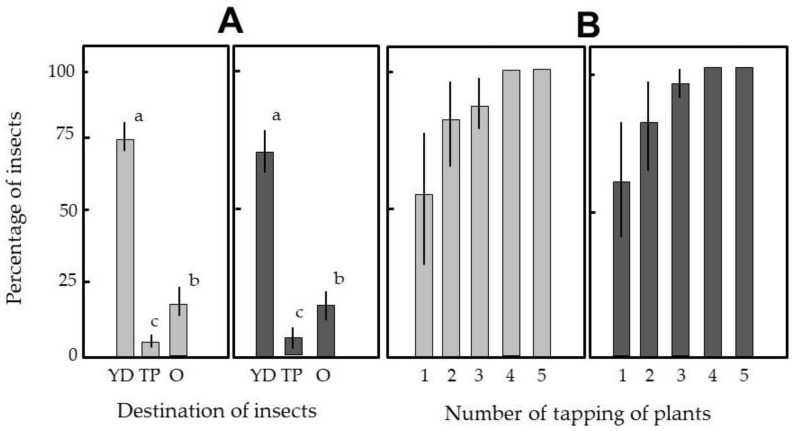
(**A**) Comparative assay of insects’ photoselective responses to yellow-colored double-charged dipolar electric field producers (DD-EFPs). Triplicated DD-EFPs (YD) were placed in front of three potted tomato plants (TP) in the closed film house; whiteflies (gray columns) and vegetable leafminers (black columns) were simultaneously blown inside the film house. At 2 h after insect release, the numbers of insects captured by the DD-EFPs, insects settled on tomato leaves, and insects remaining on other surfaces (O; floor, wall, or ceiling of the film house) were counted. (**B**) Comparison of the numbers of test insects attracted to DD-EFPs from plant leaves after single or repeated plant tappings. Insects were transferred onto tomato leaves and the leaves were tapped to cause the insects to fly from the plants. The numbers of insects captured by the DD-EFPs were counted after each tapping operation. Twenty insects were used for each species, and the means and standard deviations were calculated from five replicates of the experiments. The letters (a–c) on each vertical column in (A) indicate significant differences (*p* < 0.05) according to Tukey’s test.

In our greenhouses, tomato plants frequently experience simultaneous attacks by whiteflies and vegetable leafminers [33]. Hence, we designed an experiment in which both whiteflies and vegetable leafminers were simultaneously introduced into the closed film house, and this facilitated movement tracking. In the first experiment, we confirmed that the yellow-colored DD-EFP effectively attracted flying insects in the film house: more than 70% of the insects introduced into the film house were captured by the present apparatus (Figure 8A). Unfortunately, however, a few insects reached the tomato plants each time. This was a significant limitation because these insects rapidly breed via repeated oviposition during their lifecycle [36].

To cope with this issue, we adopted a plant-tapping method by which insects settling on leaves were forced to fly away from the plant. However, they then landed on the original or adjacent plant. These insects were expected to be attracted to the yellow-colored DD-EFP before reaching the plant. The advantage of the present method was that the apparatus could be set close to the plants because the electrified S-PVC tubes were not sticky. This close proximity of yellow-colored DD-EFPs to the plants was effective for attracting insects that had been forced to fly away (Figure 8B). Indeed, considerably high numbers of insects were attracted by a single tapping operation: in two insect species, four or five consecutive tappings were sufficient to transfer all insects from the plants to the DD-EFPs. In the last experiment, the introduction of insects into the film house was followed by five plant tappings. This experiment confirmed that the DD-EFPs were able to lure and capture all insects, including a few whiteflies that stayed on the ceiling of the film house.

The results of this study were as follows: (1) the colored water in the insulator tube conducted electricity to electrify the insulator tube via dielectric polarization; (2) a pair of insulator tubes oppositely electrified with charged colored water formed a DD-field in the space between opposing tubes; (3) the DD-field exerted sufficient force to capture insects that entered the field; (4) the tubes containing yellow-colored water successfully lured phototactic insects (e.g., whiteflies, vegetable leafminers, and western flower thrips) into the DD-field of the apparatus for their capture; and (5) the yellow-colored DD-EFP placed close to the plants preferentially attracted flying insects to the apparatus, or moved the insects settling on the plants to the apparatus via a plant-tapping operation. The present study provides an experimental basis for developing a simple and promising electrostatic apparatus to attract phototactic insect pests.

Lastly, we outlined some of the next steps for the future work in developing the present apparatus for more beneficial practical use. The main focus is to combine the present apparatus with other existing devices. The first option is the use of semiochemicals, such as aggregation pheromone or kairomone [37], to improve capture of thrips. Another option is the simultaneous use of ultraviolet rays. Many insects are attracted to certain ultraviolet frequencies [22], and this approach might reduce the need to tap plants so that insects that have landed could fly off to the trap. Further studies are essential to assess the impact of the yellow DD-EFP traps on beneficial insects such as parasitoid wasps, syrphid flies, and pollinators such as bumble bees. The attachment of a coarse screen to the DD-EFP is a possible approach to protect larger beneficials such as syrphids and bumble bees, as outlined by Chen et al. [38]. The present system could be incorporated into other greenhouse operations such as heating, cooling, or watering, by which the DD-EFP might be more practical and beneficial.

## 4. Conclusions

The DD-EFP is a simple and effective physical apparatus designed to lure and capture phototactic insect pests. Based on electrostatic principles, it generated an attractive force and inherent photoselectivity toward the target insects. Negative and positive charging of colored water filled in the transparent insulator tubes was essential for providing the tubes with the color required to lure phototactic insects, and for producing an oppositely electrified tube pair to form the DD-field for capturing insects. The DD-EFP could be placed close to the plants because of its non-sticky surface, to which the pests were effectively lured and trapped.

## Figures and Tables

**Table 1:** Percentages of test insects captured by oppositely electrified soft polyvinyl chloride (S-PVC) tubes of the double-charged dipolar electric field producer (DD-EFP) containing charged water with and without watercolors.

DD-EFP Used	Test Insect	Negative and Positive Voltage (kV) Applied to S-PVC-Tubes						
		0.2	0.4	0.6	0.8	1	1.2	2
Non-colored	Whiteflies	0	21.2 ± 7.5 a	68.5 ± 2.5 a	100 a	100 a	100	100
	Western flower thrips	0	9.5 ± 0.8 b	38.7 ± 1.5 b	82.5 ± 2.5 b	100 a	100	100
	Vegetable leafminers	0	1.5 ± 0.09 c	22.8 ± 1.7 c	64.6 ± 4.3 c	83.4 ± 1.8 b	100	100
Yellow-colored	Whiteflies	0	19.9 ± 3.3 a	61.6 ± 1.8 a	100 a	100 a	100	100
	Western flower thrips	0	8.8 ± 0.7 b	30.9 ± 2.1 b	85.1 ± 1.3 b	100 a	100	100
	Vegetable leafminers	0	1.9 ± 0.1 c	20.8 ± 0.7 c	69.6 ± 2.3 c	84.5 ± 2.2 b	100	100
Blue-colored	Whiteflies	0	20.3 ± 4.5 a	68.5 ± 2.5 a	100 a	100 a	100	100
	Western flower thrips	0	9.2 ± 0.4 b	38.7 ± 1.5 b	83.7 ± 2.2 b	100 a	100	100
	Vegetable leafminers	0	1.2 ± 0.09 c	24.1 ± 1.6 c	66.8 ± 2.3 c	87.4 ± 2.2 b	100	100
Red-colored	Whiteflies	0	21.9 ± 7.5 a	62.8 ± 1.9 a	100 a	100 a	100	100
	Western flower thrips	0	8.9 ± 1.1 b	38.7 ± 1.5 b	86.5 ± 0.5 b	100 a	100	100
	Vegetable leafminers	0	1.1 ± 0.08 c	21.9 ± 2.4 c	62.5 ± 3.9 c	82.9 ± 1.4 b	100	100

Twenty insects were used for each species and each DD-EFP. The means and standard deviations were calculated from five replicates of the experiments. The letters (a–c) on each vertical column of means indicate significant differences (*p* < 0.05) according to Tukey’s test.

## Data Availability

The data presented in this study are available in Supplementary Materials.

## Supplementary Materials

The following are available online at https://www.mdpi.com/article/10.3390/insects12110960/s1, Table S1: Potential difference at various points of soft polyvinyl chloride tube (S-PVC-T) electrified with watercolor-free and watercolor-containing water negatively or positively charged with different voltages, Video S1: Capture of test insects (whiteflies, vegetable leafminers, and western flower thrips) by the yellow-colored double-charged dipolar electric field producer (DD-EFP) charged with ±1.2 kV via (A) direct and (B) two-step methods.
