# Supplementary material for: Developing a Phototactic Electrostatic Insect Trap Targeting Whiteflies, Leafminers, and Thrips in Greenhouses
Source: Insects. 2021 Oct 21;12(11):960. doi: 10.3390/insects12110960 (PMC8622956; doi:10.3390/insects12110960)
Supplement: Supplementary file 1 [file insects-12-00960-s001.zip › Table S1.pdf]

Table S1. Potential differences at various locations along a soft polyvinyl chloride tube (S-PVC-T) electrified with watercolor-free and watercolor-containing water that had been negatively or positively charged with different voltages.

**Water in S-PVC-T**	**Negative or positive voltage (kV) applied to water in S-PVC-T**	**Potential difference (kV) of S-PVC-T at points of**							**Water in S-PVC-T**	**Negative or positive voltage (kV) applied to water in S-PVC-T**	**Potential difference (kV) of S-PVC-T at points of**						
		**1**	**5**	**10**	**20**	**39**	**40**	**80** [a]			**1**	**5**	**10**	**20**	**39**	**40**	**80** [a]
Water containing no waterclour	-1	1.00	1.00	1.00	1.00	1.00	0.99	0.98	Water containing blue waterclour [b]	-1	1.00	1.00	1.00	1.00	1.00	0.99	0.98
	1	1.00	1.00	1.00	1.00	1.00	0.99	0.98		1	1.00	1.00	1.00	1.00	1.00	0.99	0.98
	-5	5.00	5.00	5.00	5.00	5.00	4.99	4.98		-5	5.00	5.00	5.00	5.00	5.00	4.99	4.98
	5	5.00	5.00	5.00	5.00	5.00	4.99	4.98		5	5.00	5.00	5.00	5.00	5.00	4.99	4.98
	-10	10.00	10.00	10.00	10.00	10.00	9.99	9.98		-10	10.00	10.00	10.00	10.00	10.00	9.99	9.98
	10	10.00	10.00	10.00	10.00	10.00	9.99	9.98		10	10.00	10.00	10.00	10.00	10.00	9.99	9.98
Water containing yellow waterclour [b]	-1	1.00	1.00	1.00	1.00	1.00	0.99	0.98	Water containing red waterclour [b]	-1	1.00	1.00	1.00	1.00	1.00	0.99	0.98
	1	1.00	1.00	1.00	1.00	1.00	0.99	0.98		1	1.00	1.00	1.00	1.00	1.00	0.99	0.98
	-5	5.00	5.00	5.00	5.00	5.00	4.99	4.98		-5	5.00	5.00	5.00	5.00	5.00	4.99	4.98
	5	5.00	5.00	5.00	5.00	5.00	4.99	4.98		5	5.00	5.00	5.00	5.00	5.00	4.99	4.98
	-10	10.00	10.00	10.00	10.00	10.00	9.99	9.98		-10	10.00	10.00	10.00	10.00	10.00	9.99	9.98
	10	10.00	10.00	10.00	10.00	10.00	9.99	9.98		10	10.00	10.00	10.00	10.00	10.00	9.99	9.98

[a] Distance (m) from the tube end connected to a peristaltic pump.

[b] 750 mg watercolor in 100 mL of water.
